# Thermodynamic mechanism in colored glass substrates of interference filters under continuous-wave laser irradiation

**DOI:** 10.1038/s41598-025-17365-8

**Published:** 2025-08-26

**Authors:** Wangqi Xue, Zhili Zhang, Pengcheng Dou, Yubin Shi, Zuodong Xu, Jiawei Wang, Jianmin Zhang

**Affiliations:** 1 Laboratory of Intelligent Control, Rocket Force University of Engineering, Xi’an, 710025 China; 2 State Key Laboratory of Laser Interaction with Matter, Northwest Institute of Nuclear Technology, Xi’an, 710024 China

**Keywords:** Continuous-wave laser irradiation, Laser-induced damage threshold (LIDT), Colored glass substrates, Thermodynamic mechanism, Parameter inversion method, Optical physics, Statistical physics, thermodynamics and nonlinear dynamics, Applied optics

## Abstract

The ablation perforation damage of double-sided coated narrow-band filters based on RG-850 colored glass under out-of-band laser irradiation is investigated. A temperature-triggered nonlinear absorption mechanism is identified where substrate absorption sharply increases beyond a critical temperature. To quantify the resulting energy deposition dynamics, the multiple reflection model is employed, revealing the absorption enhancement by partial-transmission/high-reflection coatings. Building on this foundation, a parameter inversion method derives the equivalent average absorption coefficient from dual-transmittance laser-induced damage threshold (LIDT) ratios, thereby establishing a LIDT predictive framework for arbitrary transmittance. Finally, finite element analyses (FEA) provide validation for the multiple reflection model and inversion method, demonstrating the coating structure’s role in absorption enhancement and successfully predicting damage thresholds across three transmittance configurations.

## Introduction

Narrow-band interference filters play a crucial role in various fields, such as multispectral imaging, light detection and ranging (LiDAR), free-space communications, and remote sensing, owing to their capability to effectively filter and separate specific spectral information of interest^1–4^. However, laser-induced damage in these filters presents a significant challenge, as filter damage or degradation can lead to catastrophic failure of entire optical systems. Extensive studies have focused on laser-induced damage mechanisms in dielectric multilayer coatings, where failure modes are determined by several factors, including pulse duration, surface quality, manufacturing defects and residual stress^5–8^. These parameters dictate the damage initiation sites (within layers, at interfaces, or near coating-substrate interfaces) and subsequent damage progression patterns^9^.

For ultrafast lasers, damage primarily arises from nonlinear absorption processes such as multiphoton absorption and multiphoton ionization, resulting in highly localized energy deposition and surface or subsurface ablation^10–15^. In contrast, long-pulse or continuous-wave (CW) lasers induce thermal accumulation in both coatings and substrates^16–18^. In such cases, the laser-induced damage threshold (LIDT) is determined by the combined effects of interference-modulated electric field distributions, typically analyzed through transfer matrix methods, and thermal diffusion dynamics^19–22^. This analytical framework also applies to local field enhancement phenomena caused by defects or inclusions^23–26^. Although these studies provide insights into coating-dominated damage mechanisms, substrate-dominated failure modes remain insufficiently investigated. When damage initiation occurs within the substrate bulk, the energy deposition mechanism transitions from interference-dominated field enhancement to bulk absorption processes governed by the substrate’s intrinsic optical and thermal properties. In this regime, the coating functions primarily as a spectral selector through its transmission/reflection characteristics rather than creating localized field modulation. This mechanistic transition becomes particularly relevant when analyzing colored glass substrates, which exhibit dual wavelength- and temperature-dependent absorption characteristics. The RG series colored glasses are widely employed as substrates for near-infrared (NIR) filters due to their strong visible-light absorption and high NIR transmittance. Although the LIDT of RG series colored glass in visible wavelengths has been documented at approximately 30 W/cm^2^^27^, the thermodynamic response of coated RG-850 substrates under NIR CW laser irradiation remains insufficiently characterized. Thermodynamic characterization of such processes typically relies on direct temperature monitoring or finite element modeling^28–30^. However, for coated components, the near-infrared filter coatings’ opacity to long-wave radiation critically impedes both experimental temperature observation and model validation, further complicating thermodynamic analysis and LIDT prediction.

In this study, we reveal a substrate-dominated ablation perforation mechanism characterized by bulk instantaneous vaporization without progressive coating damage, driven by temperature-triggered nonlinear absorption intensification beyond ~ 200 °C. We develop an energy-deposition-based methodology comprising three synergistic parts: (1) a multiple reflection model quantifying coating-modulated energy deposition; (2) a parameter inversion technique extracting the equivalent average absorption coefficient from dual-transmittance LIDT ratios and enabling LIDT prediction for arbitrary transmittance configurations; (3) finite element modeling incorporating the inverted absorption coefficient for cross-verification and detailed thermodynamic analysis. This research architecture is extensible to LIDT prediction and thermodynamic analysis for similar substrate-dominated coated components.

## Experiments

### Sample parameters

**Fig. 1:**
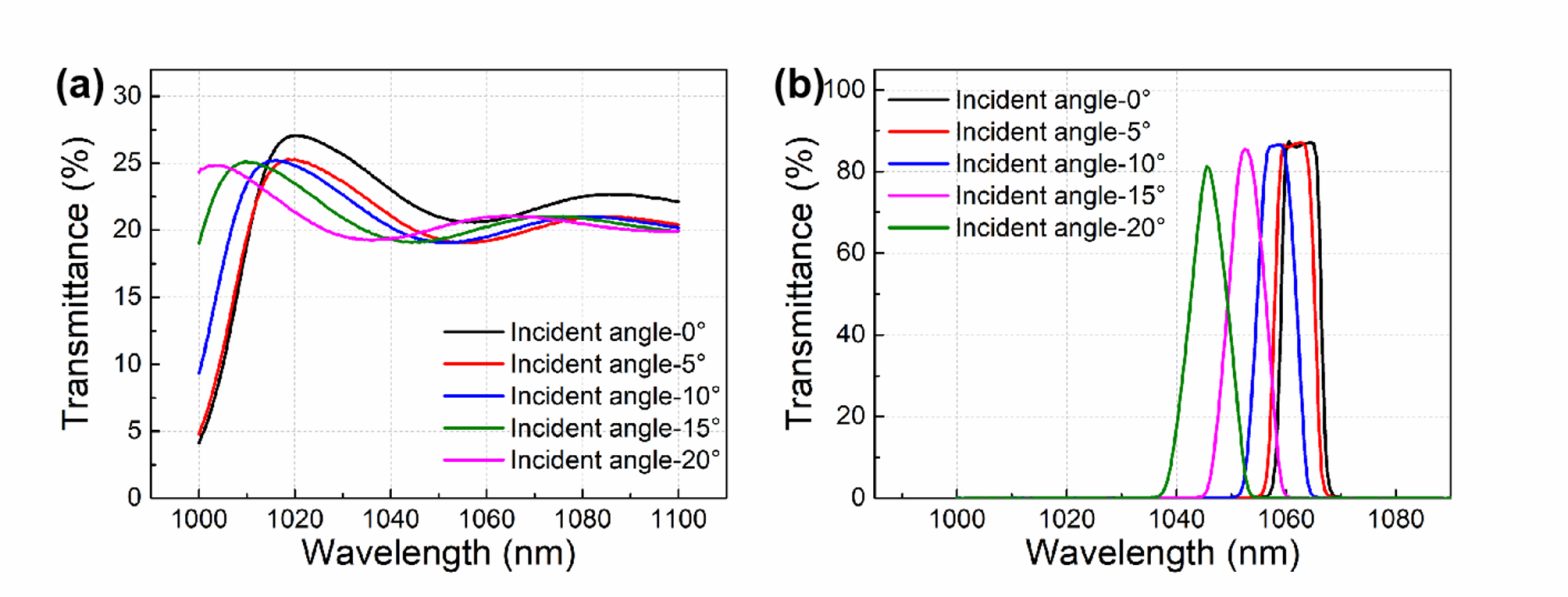
Transmittance results of the filter with unilateral coating of one type of film. (**a**) Represents the partial transmission film, transmitting at a constant value of approximately 20% within the wavelength range of 1040–1100 nm; (**b**) represents the filter film, selectively transmitting around the central wavelength of 1064 nm.

The samples utilized in the laser irradiation experiment are commercial narrow-band interference filters whose diameter and thickness are 40 mm and 3 mm, respectively. One side of the RG-850 colored glass filter is coated with a dielectric partial transmission film, which maintains a constant transmittance from 1040 nm to 1100 nm, as shown in Fig. 1a. Three groups of samples with different transmission/reflection (T/R) ratios of 20:80, 40:60 and 90:10, respectively, were available for the laser damage experiment. The transmittances of three types of partial transmission films are not sensitive to the incident angle, only the transmittance results of the sample with T/R ratio of 20:80 are shown for clarity. The other side is coated with a filter film, which exhibits a transmittance peak centered at 1064 nm, with a full width at half maximum (FWHM) of approximately 7.5 nm, as shown in Fig. 1b. The performance of the filter film remains consistent across all three groups. The transmittance peak of the filter film displays a blue shift and a sharper peak shape as the incident angle increases.

### Experiment setup

**Fig. 2:**
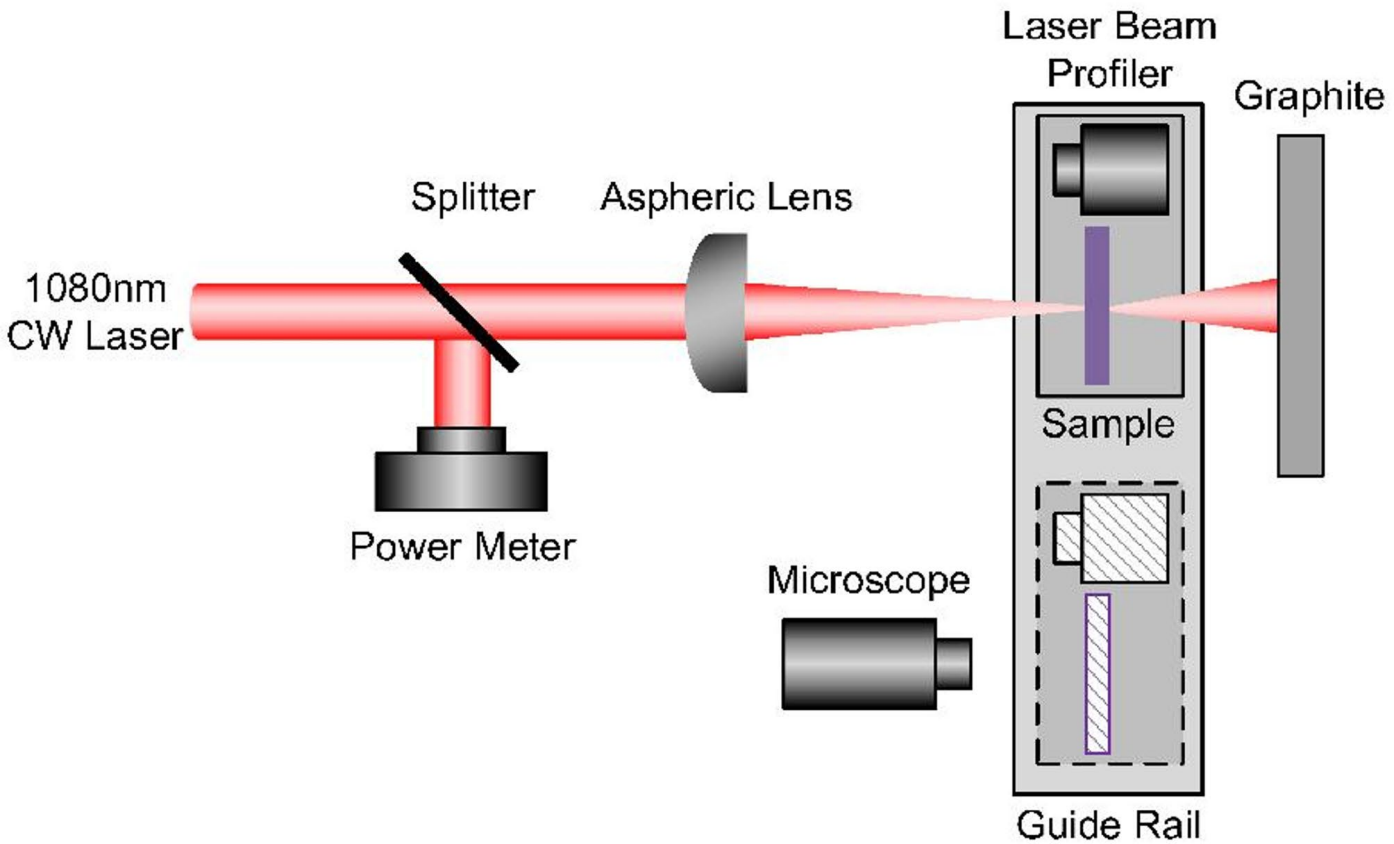
The experiment setup for laser irradiation of the filter sample.

The experiment setup for laser irradiation is shown in Fig. 2. A Yb-doped fiber CW laser is used as the heating source whose maximum average power was rated for 2 kW. The collimated beam at the laser exit has a diameter of 1 cm. A beam splitter is used to measure the laser power. The wavelength is 1080 nm and the diameter of the laser beam is approximately 1 mm after it is focused by a lens of *f*  =  2000 mm focal length on the filter sample at normal incidence. Based on geometric optics calculations, the diameter of the beam spot on the rear surface of the sample after focusing is only approximately 1.5% smaller than that on the front surface. Consequently, the difference in beam diameters between the front and rear surfaces can be negligible. The irradiation time is 5 s. The three groups of filters mentioned above are used as the irradiation samples. The laser spatial mode is Gaussian (TEM_00_ mode), which is measured by a LT665 Beam Profiling Camera (OPHIR). A microscope (Infinity2-2 M) is used to make sure the camera and sample in the same plane and a guide rail is used to switch the position. Meanwhile, the sample can be moved to the microscope through the guide rail to observe the damage state after every single irradiation. The filters damage experiment is performed at least 3 shots for each laser power setting using the 1-on-1 method. The distance between two irradiation spots is greater than twice of the radius according to ISO 21,254 ^31^.

## Results and discussion

### Damage results

In this experiment, the LIDT is expressed as power per spot size (W/cm). In long pulse or CW regime, the laser damage is mainly caused by thermal effect. When the heat conduction outside the irradiation area is non-negligible, the illuminated area is regarded as the linear source. In this way, the temperature rise required for melting or stress failure is given by the following formula^32^,

1 $$dT=\frac{{P\alpha }}{{2\rho Cr\pi D}}$$

where *P* is the power, *α* is the absorption coefficient, *ρ* is the density, *C* is the heat capacity, *r* is the radius of beam spot, *D* is the diffusion rate. The temperature rise required for damage is proportional to power per spot size, indicating that the term linear power density (LPD) is applicable to any beam diameter^33^.

**Fig. 3:**
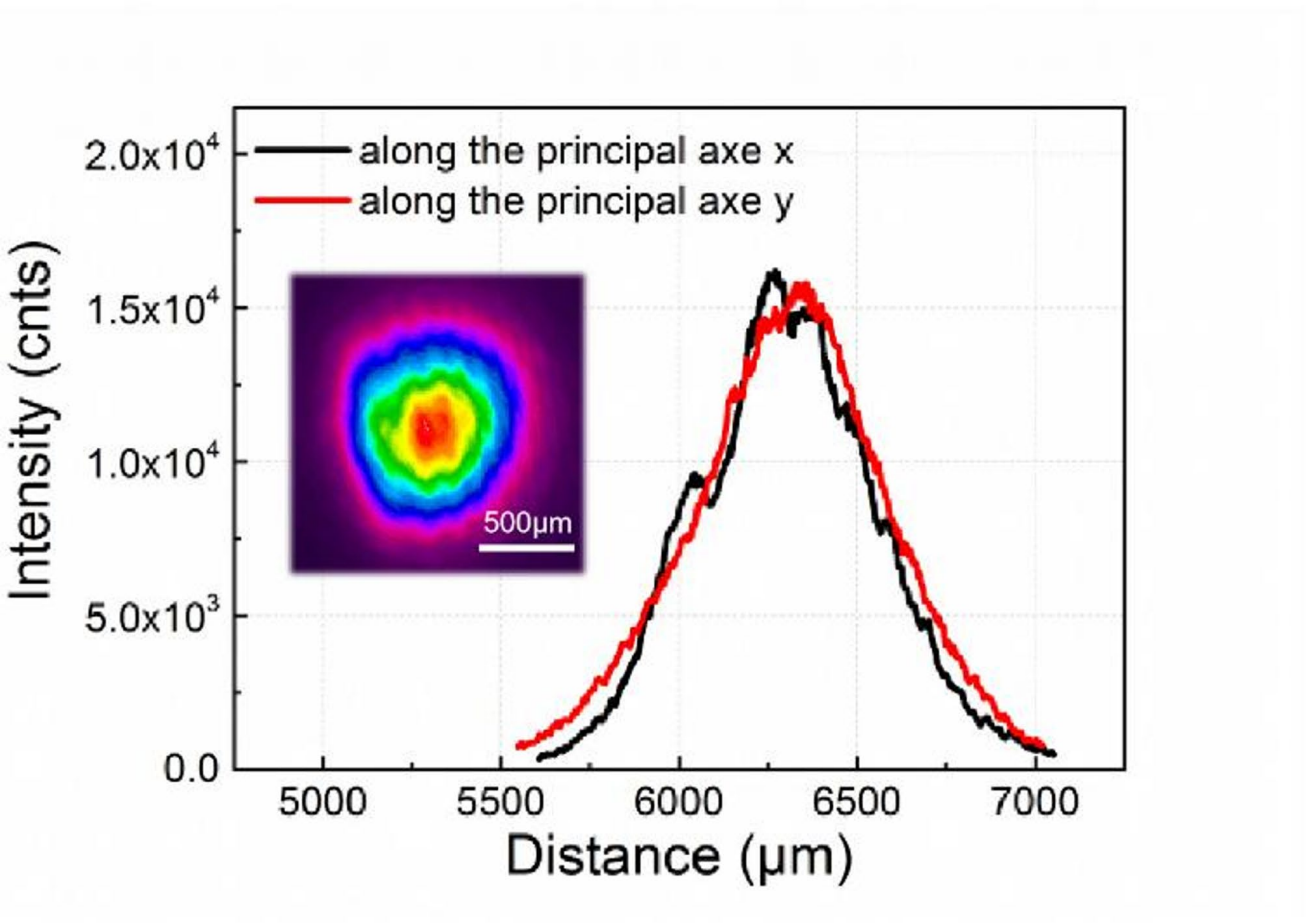
The beam spot image and the power density distribution in a cross-section along the principal axes x and y.

The beam spot images and the power density distribution in a cross-section along the principal axes x and y are illustrated in Fig. 3. By adjusting the position of the lens, the diameter of the beam spot on the sample surface is measured to be 1.06 mm. The diameter of beam spot is determined using the second-order moments of the power density distribution according to ISO 11146^34^.

**Fig. 4:**
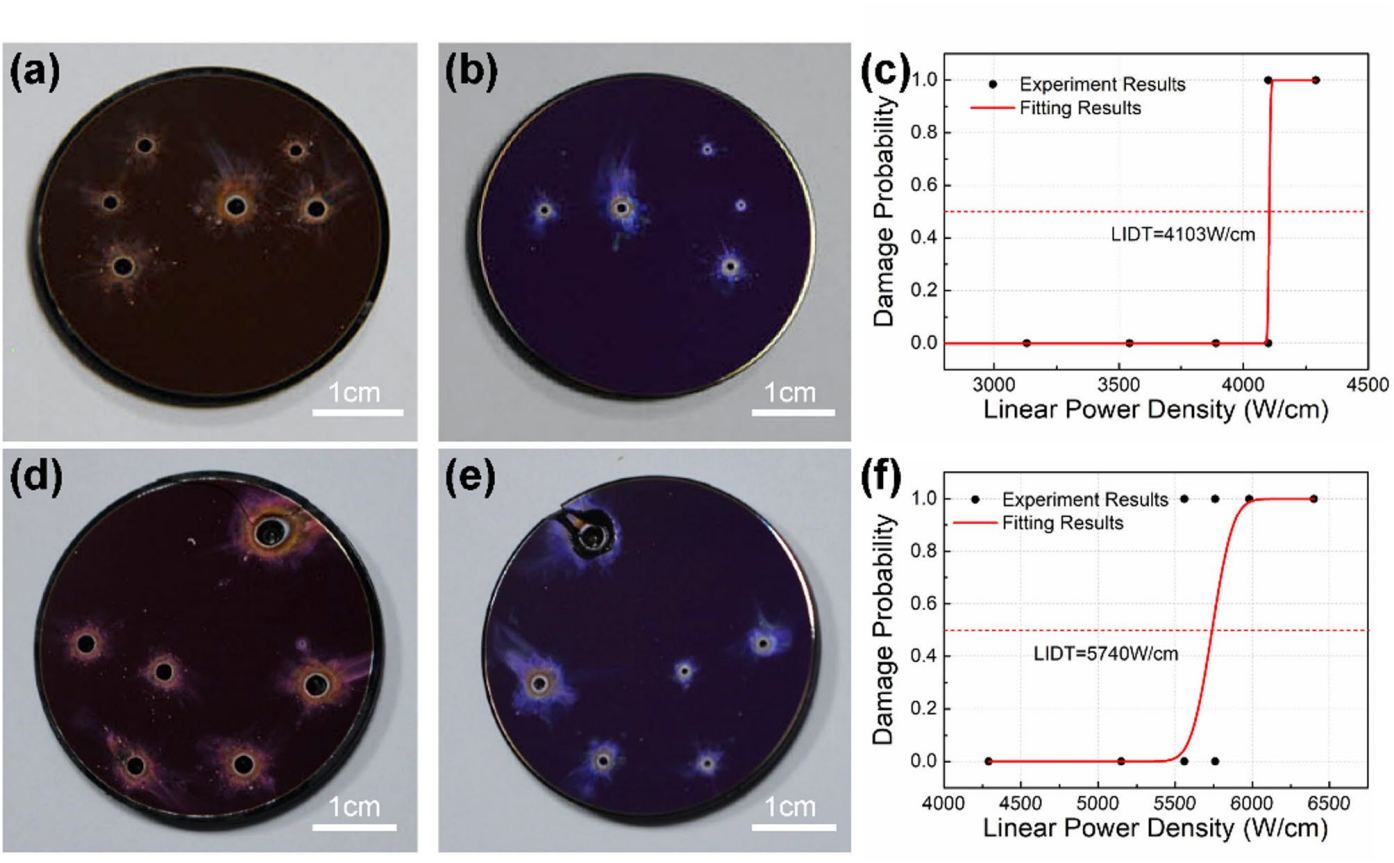
Typical damage images and the fitting LIDT results of the filter. (**a**,**b**) Showed the partial transmission side and filter side, respectively, of the filter sample with the T/R ratio of 40:60; (**d**) and (**e**) showed the partial transmission side and filter side, respectively, of the filter sample with the T/R ratio of 20:80; (**c**) and (**f**) showed the fitting results of the filter samples with the T/R ratios of 40:60 and 20:80, respectively.

In the experiment, the laser beam is incident on the filter at normal incidence from the partial transmission side. Figure 4 displays the typical damage images of the filters. Figure 4a,b show the partial transmission side and filter side, respectively, of the filter sample with the T/R ratio of 40:60, while Fig. 4d,e show the partial transmission side and filter side, respectively, of the filter sample with the T/R ratio of 20:80. In all three groups of samples, the damage process is identified as ablation perforation, and the damage morphology of the filter samples with different T/R ratios is similar. The damaged area on the back surface is slightly smaller than that of the front surface. Inside the filter, a circular truncated cone ablation hole was formed, extending throughout the whole filter. Additionally, spattered ablative products can be seen surrounding the damage area. If the film of the optical component is initially damaged, the subsequent formation of ablation holes typically follows a cumulative process. During the irradiation, one would expect to observe the holes emerging on the surface and gradually expanding inward, usually accompanied by a continuous ablation sound. In such cases, by reducing the irradiation power, it should be possible to observe a state where the film incurs damage while the substrate remains undamaged, such as displaying a typical ‘onion-ring’ damage^9^. However, the experiments were repeated multiple times, as long as ablation damage occurred, the ablation holes formed instantaneously, accompanied by a transient ablation sound. After hearing the sound, we halted the light emission immediately and observed ablation holes had already penetrated the entire thickness of the substrate. In contrast, when no ablation damage occurred, microscopic observation failed to discern any film damage. The inherently low absorption coefficient of RG-850 colored glass at ambient temperature results in the entire cylindrical region illuminated by the laser beam undergoing heating. Once the temperature within this cylindrical volume uniformly reaches the critical transformation temperature, damage occurs rapidly within a short timescale. We infer that the absorption coefficient of RG-850 colored glass increases with rising temperature, which enables the deposited energy to sufficiently elevate the temperature of the illuminated region to the transformation point. In addition, the increase in absorption coefficient leads to a temperature gradient along the thickness of the substrate, with the temperature near the partial transmission side (incident surface) slightly higher compared to the filter side. This provides a reasonable explanation for the observation that the ablation holes on the partial transmission side are marginally larger than those on the filter side. These findings demonstrate that ablation damage follows a sudden, threshold-like failure mechanism, suggesting that the substrate incurs damage prior to discernible film damage, and that the principal factor lies within the thermal process associated with the absorption of laser energy by the substrate. Also, when the laser irradiated from the dielectric filter film side, no damage occurred even at twice the power density (9790 W/cm). This is because the irradiation is reflected back into the atmosphere by the first surface of the filter coatings, which can be regarded as high reflection coatings, leaving only a small percentage to reach the substrate.

The irradiations were repeated at least three times at each laser power setting. The damage probability is described using a binomial distribution^35^with “1” and “0” representing the damaged and undamaged states, respectively. The power density at which the damage probability reached 50% is considered as the damage threshold. The typical fitting results of the filter samples with the T/R ratios of 40:60 and 20:80 are shown in Fig. 4c,e, respectively. All the LIDT results are presented in Table 1.

**Table 1:** LIDT results of filters with different T/R ratios.

Irradiation surface	T/*R* ratio	Irradiation time/(s)	Beam diameter/(mm)	LIDT/(W/cm)
Partial transmission side	20:80	5	1.06	5740
Partial transmission side	40:60	5	1.06	4103
Partial transmission side	90:10	5	1.06	3872
Filter side	–	5	1.06	> 9790

### Parameter inversion and LIDT prediction

**Fig. 5:**
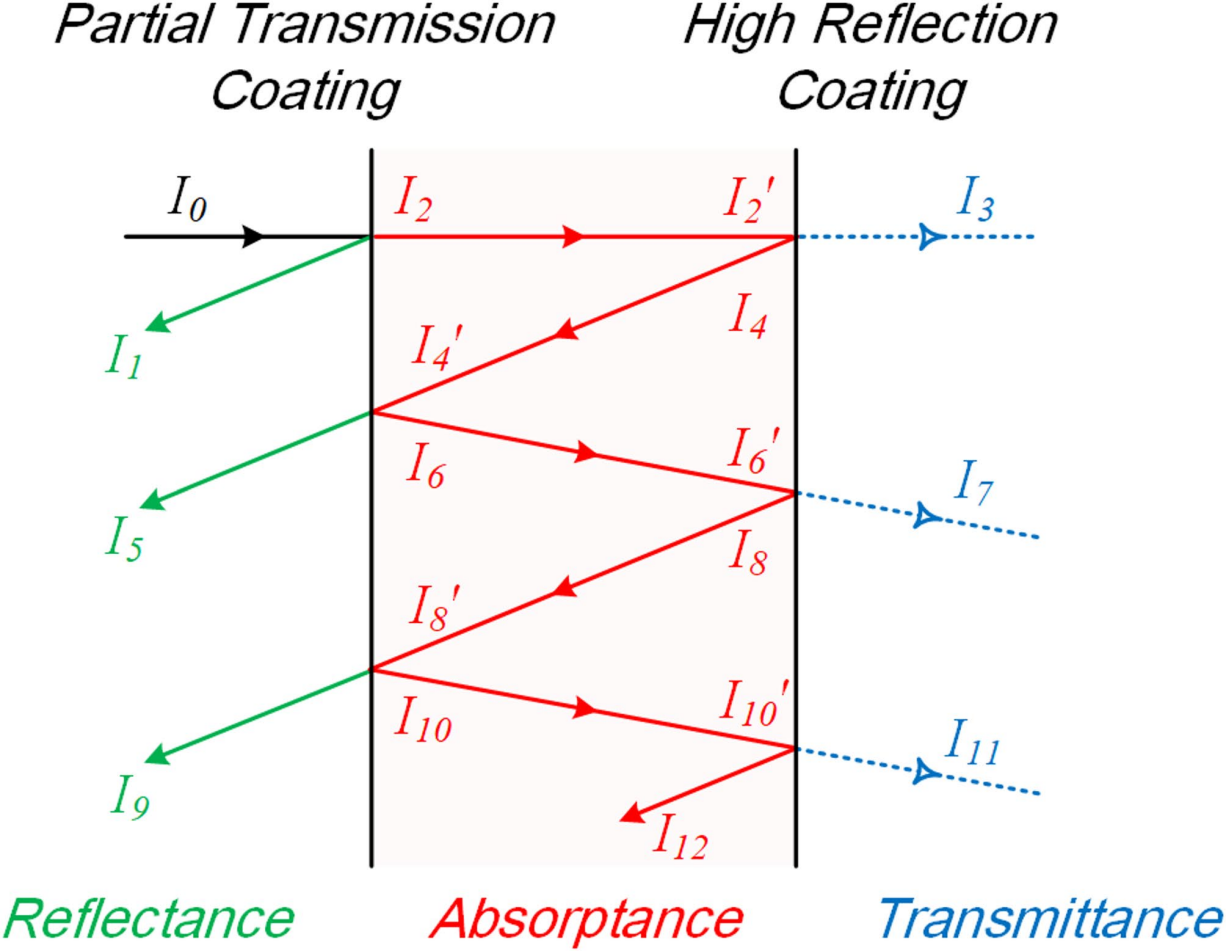
Schematic of reflectance, absorptance and transmittance of the multiple reflection model.

Generally, dielectric films are not absorbing for CW lasers and exhibit high damage thresholds. Therefore, the partial transmission film on the front surface of the sample merely alters the T/R ratio, and the filter film on the rear surface behaves as a high reflection film at 1080 nm laser. The ablation damage of the filter is attributed to the heat absorption by the substrate. Typically, the relationship between heat deposition and temperature rise is expected to be linear. However, our experiment, which involved three different types of filters with the transmittances of 20%, 40% and 90%, demonstrated a non-linear relationship between LIDT and transmittance, further suggesting that the relationship between heat deposition and front surface transmittance was also non-linear. Therefore, the multiple reflection model is employed to explore the energy deposition process. The model assumes that the filter sample consists of three parts, the partial transmission coating, the colored glass substrate and the high reflection coating. As shown in Fig. 5, the laser incident from the partial transmission side is divided into three parts after passing through the optical element: reflection, transmission, and absorption^9^. The angled beam paths are schematic only, and that the actual multiple reflections and transmissions are at normal incidence.

The partial transmission and high reflection coating structure induce multiple reflections of the incident laser between the front and back surfaces of the filter, thereby altering the absorption behaviors of the substrate. The reflectance of the high reflection coating is approximated as 1 and the transmission part is ignored. Therefore, the absorption part is expressed as a function of the absorption coefficient, sample thickness, the reflectance and transmittance of the partial transmission coating. The absorbed energy (*A*) is described by the following equation,

2 $$\begin{gathered} A={I_2} - {I_2}^{\prime }+{I_4} - {I_4}^{\prime }+{I_6} - {I_6}^{\prime }+ \cdots \\ ={I_0} - \left( {{I_1}+{I_5}+{I_9}+ \cdots } \right) - \left( {{I_3}+{I_7}+{I_{11}}+ \cdots } \right) \\ ={I_0} - \left( {{I_0}{R_1}+{I_0}{T_1}^{2}{e^{ - 2d\alpha }}+{I_0}{T_1}^{2}{R_1}{e^{ - 4d\alpha }}+ \cdots +{I_0}{T_1}^{2}{R_1}^{{n - 1}}{e^{ - 2nd\alpha }}} \right) - \left( {{I_0}{T_1}{R_1}^{n}{e^{ - 2nd\alpha }}} \right) \\ ={I_0}\left( {1 - \left( {{R_1}+{T_1}^{2}{e^{ - 2d\alpha }}+{T_1}^{2}{R_1}{e^{ - 4d\alpha }}+ \cdots +{T_1}^{2}{R_1}^{{n - 1}}{e^{ - 2nd\alpha }}} \right) - \left( {{T_1}{R_1}^{n}{e^{ - 2nd\alpha }}} \right)} \right) \\ \end{gathered}$$

where *I*_*0*_ is the power of the incident laser and assumed to be equal to “1” in the following calculations, *α* is the absorption coefficient, *d* is the sample thickness, *n* is the number of reflections, *R*_*1*_ and *T*_*1*_ are the reflectance and transmittance of the partial transmission coating, respectively.

**Fig. 6:**
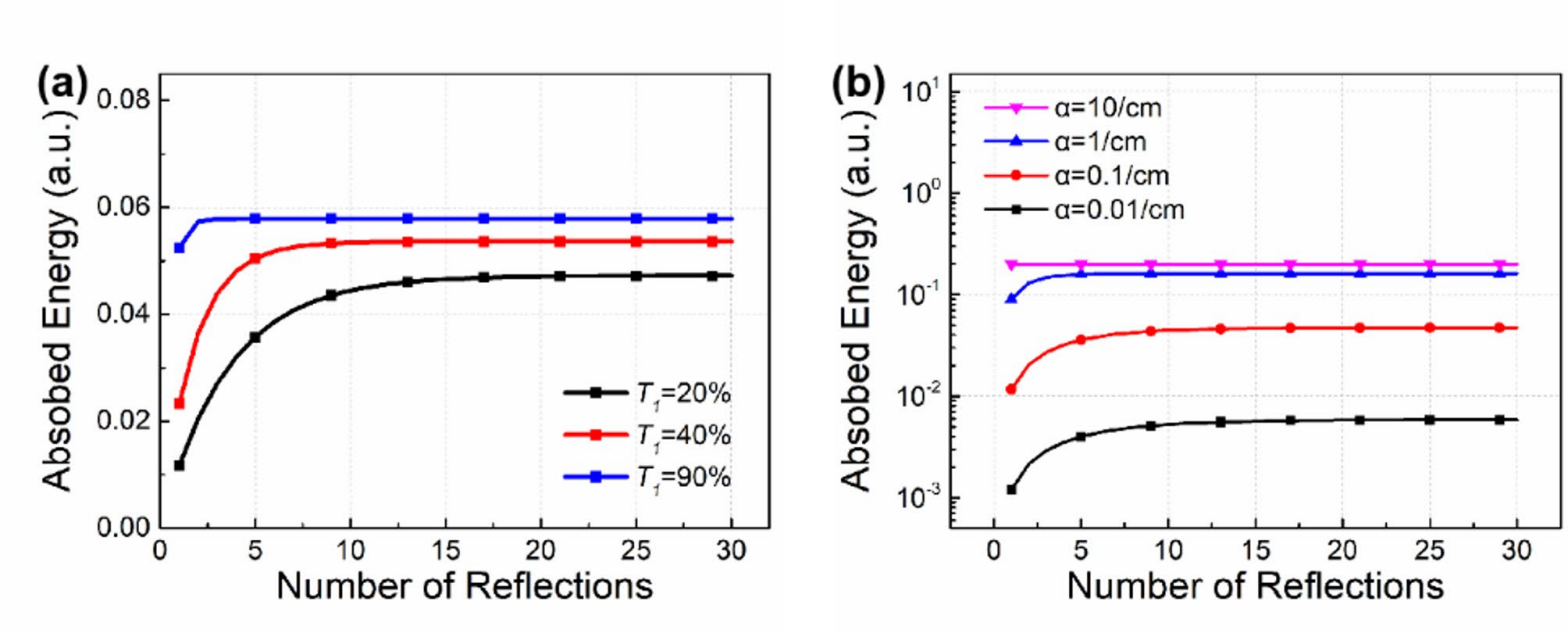
The relationship between absorbed energy and number of reflections. (**a**) was under different transmittance conditions; (**b**) was under different absorption coefficient conditions.

Due to normal incidence, laser irradiation is absorbed in a cylinder passing through the substrate along the axis of the beam spot. In this case, the value of *n* should be taken as infinity, however, as the number of reflections increases, the laser energy transmitted through the substrate gradually decreases. It is found that the number of reflections required to achieve a stable energy absorption by the substrate exceeds 20 when *T*_*1*_ equals to 20%. As the transmittance increases, this value decreases. Similarly, with an increase in the absorption coefficient, a smaller number of reflections is necessary to attain a stable energy absorption by the substrate, as shown in Fig. 6. This is because a smaller value of *T*_*1*_ or *α* implies that the incident laser needs to deposit energy over a longer distance, namely more reflections. By undergoing these additional reflections, each time the laser reaches the partial transmission coating, more energy is transmitted out of the coating and exits the substrate. Consequently, when the absorption eventually reaches a steady value, the total energy absorbed by substrate decreases accordingly with the decrease of *T*_*1*_ or *α*.

**Fig. 7:**
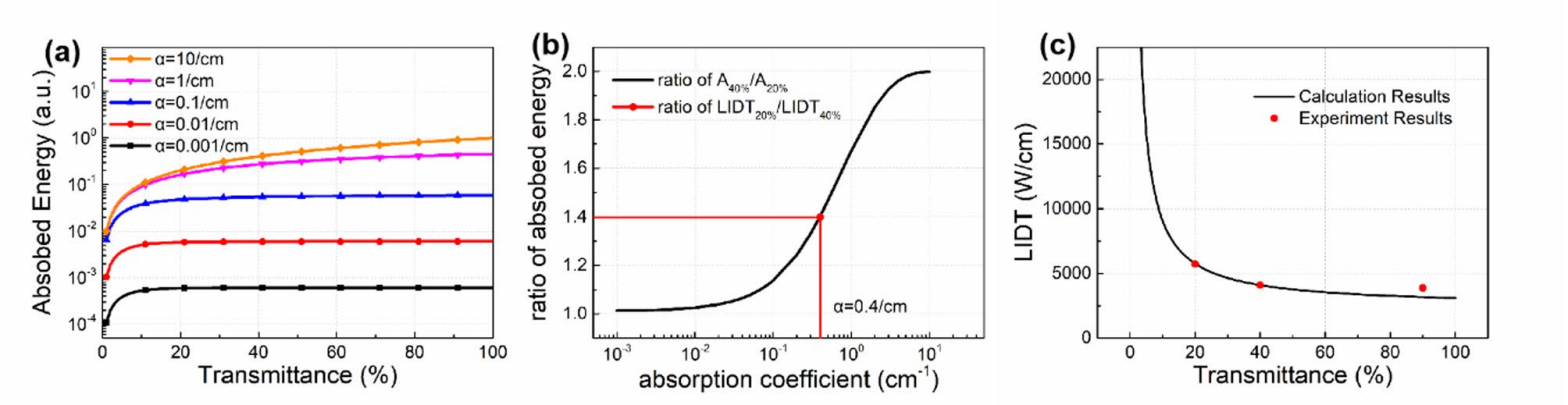
Absorption coefficient inversion and LIDT prediction. (**a**) Was the relationship between energy absorbed by the colored glass substrate and transmittance of the partial transmission film for different absorption coefficients of the colored glass; (**b**) was the parameter inversion result of the absorption coefficient; (**c**) showed the comparison between calculated and experimental LIDT results.

Due to the uncertainty in the absorption coefficient of the substrate, a series of curves are obtained by varying the absorption coefficient to illustrate the relationship between *A* and *T*_*1*_, as shown in Fig. 7a. It is found that the absorbed energy *A*(*T*_*1*_, *α*) is partially increasing with respect to both *T*_*1*_ and *α*, thus, for any two constant values *T*_*1a*_ and *T*_*1b*_, the ratio of *A*(*T*_*1a*_, *α*) and *A*(*T*_*1b*_, *α*) remains an increasing function with respect to *α*. Therefore, a data analysis method based on the experimental LIDT results is proposed to achieve two purposes: parameter inversion of the absorption coefficient of the substrate, and prediction of the LIDT values of filters with different *T*_*1*_ values.

Specifically, two distinct transmittance values of 20% and 40% are selected to keep the consistency with the experimental setup. The absorbed energy at transmittance of 20% and 40% is denoted as *A*_20%_ and *A*_40%_, respectively. Similarly, the damage thresholds of the filter at transmittance of 20% and 40% are denoted as LIDT_20%_ and LIDT_40%_, respectively. As shown in Fig. 7b, the *A*_40%_/*A*_20%_ ratio exhibits a monotonic increase as the absorption coefficient increased. Meanwhile, the absorbed energy and LIDT are inversely proportional, indicating that the trend in LIDT_20%_/LIDT_40%_ ratio should be consistent with that of *A*_40%_/*A*_20%_ ratio. Therefore, the experimental values of the LIDT ratio can be utilized to determine the absorption coefficient. According to the experimental results in Table 1, the LIDT_20%_/LIDT_40%_ ratio is approximately 1.4. Then, the absorption coefficient corresponding to *A*_40%_/*A*_20%_ ratio of 1.4 is determined from Fig. 7b, which results in an inversion result of 0.4/cm. Moreover, when the absorption coefficient reaches a considerable magnitude (approximately 10/cm), the substrate absorbs almost all the energy without requiring reflections between front and back surfaces to occur. In this case, the partial transmission and high reflection coating structure has a minimal effect on the energy absorption behaviors. Consequently, the absorbed energy exhibits a linear relationship with transmittance, which explains why the *A*_40%_/*A*_20%_ ratio approaches approximately 2.

The absorption coefficient obtained through parameter inversion is then substituted back into formula 2. However, a discrepancy persists between the actual LIDT values and the reciprocal of the absorbed energy (1/*A*) taking the form of a constant product term which is related to the thermodynamic parameters. The constant term is then represented using the known LIDT_40%_ and *A*_40%_ results. In this way, the following equation can be utilized to calculate the LIDT results for any given transmittance values,

3 $$LID{T_{T\% }}=\frac{{LID{T_{40\% }}}}{{{A_{40\% }}}} \times \frac{1}{{{A_{T\% }}}}$$

To evaluate the accuracy of the calculation, the discrepancy between the calculated and experimental results of LIDT is analyzed, as shown in Fig. 7c. The deviation between the calculated and experimental values of LIDT_20%_ is minimal because the proportional relationship between LIDT_20%_ and LIDT_40%_ has been incorporated in the calculation process. Subsequently, an additional experiment was conducted to obtain the experimental value of LIDT_90%_, which is already shown in Table 1. As a result, the calculated value is found to be 17.8% lower than the experimental value, falling within an acceptable range of deviation.

It’s worth noting that the RG-850 colored glass features a cut-off edge of 850 nm, displaying high transmittance for near-infrared light beyond this wavelength. At the wavelength of 1080 nm, the absorption is found to be less than 1% at room temperature, indicating an absorption coefficient of the substrate less than 0.034/cm. However, the inversion result is an order of magnitude higher, with a value of approximately 0.4/cm, which is attributed to the rapid increase in the absorption coefficient with the rise in temperature. The inversion result, therefore, represents an “averaged” absorption coefficient of the entire heating process. In our subsequent work, a finite element model including the thermodynamics module will be developed to simulate the complete damage process of the filter. We anticipate achieving a more accurate representation of the actual temperature rise process during the simulation, by utilizing the inversion result of the absorption coefficient instead of relying on the room temperature value.

### Finite element model construction and thermal process analysis

To further validate the numerical model, we developed two types of finite element models. First, an uncoated substrate model was established to accurately describe the energy absorption and thermal conduction processes during temperature evolution, thereby verifying the correctness of model parameters. Subsequently, a coated component model was constructed to analyze the energy deposition enhancement induced by coating structures, demonstrating the feasibility of predicting damage thresholds for three transmittance variants (20%/40%/90%) using the equivalent mean absorption coefficient.

**Fig. 8:**
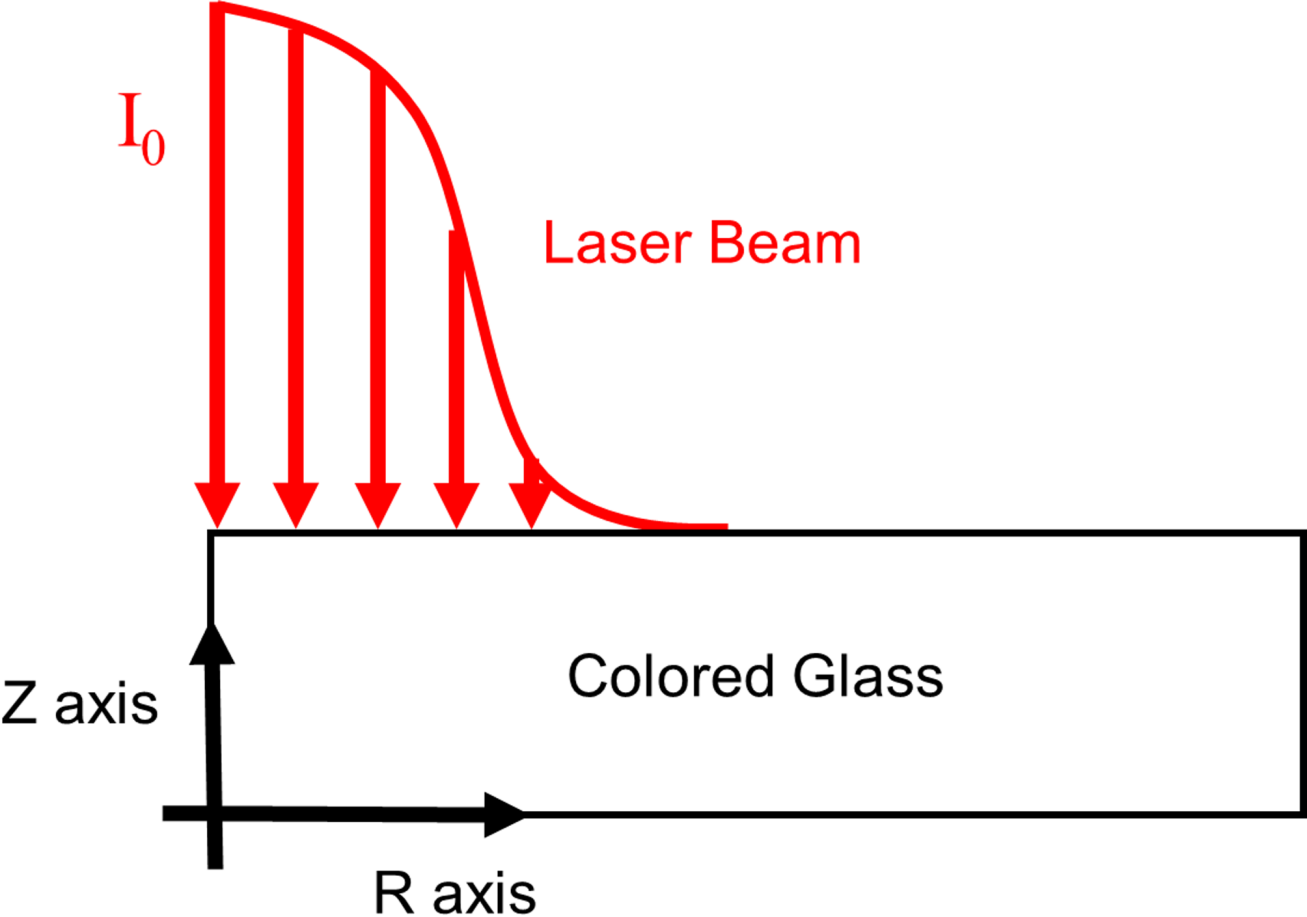
The diagram of computational domain, laser beam for simulation.

A 2D axisymmetric finite element model was implemented, as shown in Fig. 8. The Z-axis represents the symmetry axis (thickness direction), while the R-axis denotes the radius direction. The laser energy profile follows a Gaussian distribution and the incident location is at the center of the sample. Temperature distribution follows the Fourier heat transfer equation:

4 $$\rho c\frac{\partial }{{dt}}T\left( {r,z,t} \right)=\frac{1}{r}\frac{\partial }{{\partial r}}\left( {rk\frac{{\partial T\left( {r,z,t} \right)}}{{\partial r}}} \right)+\frac{\partial }{{\partial z}}\left( {k\frac{{\partial T\left( {r,z,t} \right)}}{{\partial z}}} \right)+Q\left( {T,r,z,t} \right)$$

where *ρ* is density, *c* and *k* indicate the heat capacity and thermal conductivity. *T* is the instant temperature, and *Q* is the heat source term that includes both laser energy deposition and surface convective/radiative losses. The *ρ*, *c* and *k* of RG-850 glass were set to 2930 kg/m^3^, 800 J/(kg K), and 1.35 W/(m K) respectively. The initial temperature and ambient temperature were both 25 °C.

**Fig. 9:**
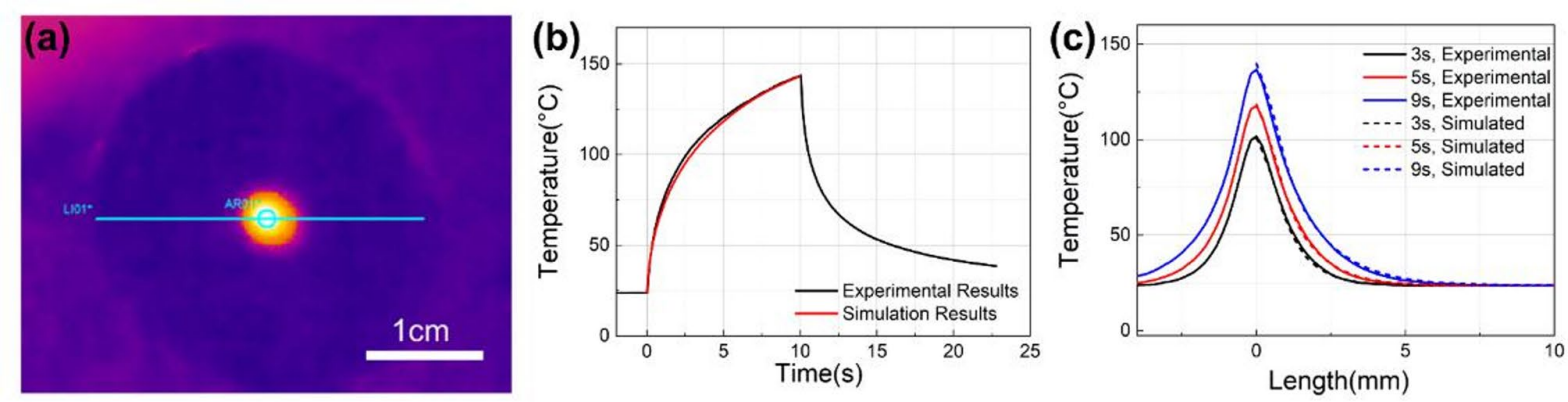
Thermal response of the uncoated RG-850 sample under laser irradiation. (**a**) Represents the thermal image captured at 1 s and illustrates the monitoring regions; (**b**) represents the maximum temperature evolution within a 1 mm diameter central monitoring region; (**c**) represents the experimental and simulated results of the surface temperature profiles along the centerline after laser irradiation at 3 s, 5 s, and 9 s.

Under irradiation conditions of 1800 W power, 1 mm spot diameter, and 10 s duration, the uncoated sample exhibited observable heating without ablation damage or surface damage. Temperature evolution was monitored using a FLIR T660 thermal imager. Figure 9a displays the thermal image at 1 s after irradiation onset, showing both the circular monitoring region (~ 1 mm diameter on the sample surface) and the linear profile measurement path. Figure 9b compares the experimental and simulated maximum temperature evolution within the circular monitoring region and, while Fig. 9c shows their corresponding temperature distributions along the linear profile path at 3 s, 5 s and 9 s, respectively. Due to the axisymmetric nature of the model, the simulation results display only half of the full cross-section. Both figures demonstrate good agreement between measurements and simulations.

**Fig. 10:**
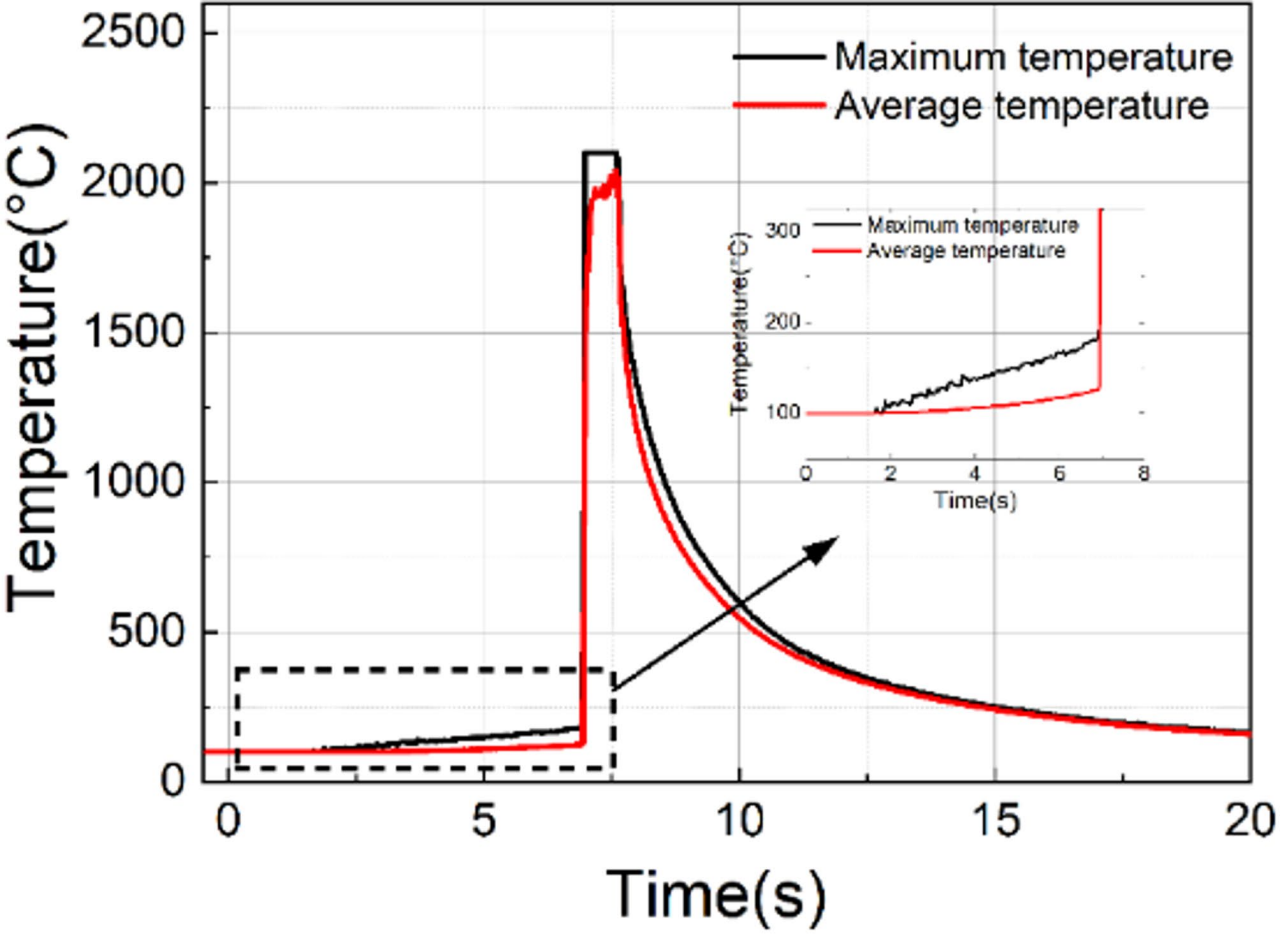
Temperature evolution during central damage of the uncoated RG-850 sample: Maximum and average temperatures within a 1 mm diameter central circular monitoring region.

When the spot diameter was reduced to 0.7 mm under identical laser power (1800 W), ablation damage occurred at approximately 7 s after irradiation initiation. Figure 10 presents the evolution of both maximum and average temperatures at the center. The damage process comprises three distinct stages including initial slow heating, nonlinear transition at 200 °C, and post-ablation behavior. During the initial irradiation stage, the temperature increased gradually until reaching approximately 200 °C. At this critical point, a sharp rise in absorption triggered rapid heating to over 2100 °C, approaching silica’s vaporization temperature of ~ 2200 °C^30^ and culminating in ablation. The thermal camera’s detection limit constrained measurements, with a minimum detectable temperature of 100 °C and a saturation temperature of 2100 °C. Nevertheless, the average temperature profile suggests the actual peak slightly exceeded the instrument’s upper limit while still capturing the essential trend. Laser emission ceased immediately after ablation initiation, resulting in rapid temperature decay.

**Fig. 11:**
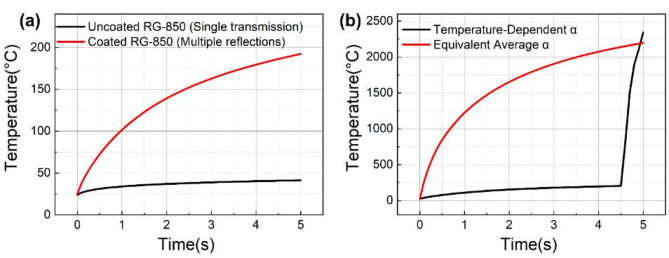
Simulation results illustrating the effect of coating structure and absorption coefficient modeling on the maximum temperature evolution. (**a**) Shows comparison of the uncoated and coated RG-850 samples at the same power density (4100 W/cm); (**b**) shows comparison for the coated sample using equivalent average absorption coefficient and temperature-dependent absorption coefficient.

For the coated component model with 2200 °C as the damage threshold, structural absorption enhancement below the critical temperature was analyzed. At 410 W incident power with 1 mm spot diameter and 5 s irradiation duration, the coated component reached 192 °C maximum temperature without significant absorption coefficient increase. This temperature rise resulted exclusively from multiple reflections in the coating structure. By contrast, under identical irradiation conditions, the uncoated substrate model with single transmission reached only 41 °C maximum temperature, as shown in Fig. 11a. These results confirm that multiple reflections substantially enhance absorption before reaching critical temperature. The complete damage mechanism originates from coating-induced multiple reflections which reduce the time to reach critical temperature at low absorption coefficients. Subsequent temperature rise to critical temperature triggers significant absorption increase, ultimately causing ablation.

Comparative analysis of temperature evolution was performed by implementing both an equivalent average absorption coefficient and a temperature-dependent absorption coefficient in the model. The simulation results using the temperature-dependent absorption coefficient reproduced the characteristic thermal evolution for 20% transmittance at 5400 W/cm: initial gradual temperature rise, abrupt transition at 200 °C, and rapid heating reaching the 2200 °C damage threshold at ~ 5 s, as shown in Fig. 11b. When applying a constant absorption coefficient of 0.17/cm, identical damage time was achieved under the same conditions. Extending this value to 40% and 90% transmittance cases while maintaining the 5 s damage time yielded corresponding thresholds of 4400 W/cm and 3900 W/cm, respectively. Validation against experimental results showed threshold deviations of − 6.3% (T = 20%), + 7.3% (T = 40%), and + 0.7% (T = 90%). Notably, while the equivalent average absorption method does not reproduce actual temperature profiles, it provides an effective and practical approach for determining damage thresholds and temporal progression in coated optical components where direct temperature monitoring is infeasible.

Finite element validation identified α = 0.17/cm as the optimal equivalent average absorption coefficient, yielding minimal LIDT prediction errors. Although this value shares the same order of magnitude with the initial 0.4/cm estimate, the deviation prompted rigorous uncertainty analysis. The overall LIDT uncertainty originates from laser spot diameter (± 3%), laser power (± 3%), and statistical fitting (± 1.5%), propagating to a 9.7% uncertainty in the LIDT_20%_/LIDT_40%_ ratio (1.4 ± 0.136), which corresponds to an equivalent average absorption coefficient range of 0.2/cm to 0.6/cm. Given the validation by finite element modeling of α = 0.17/cm, we directly input this value into the multiple reflection model, yielding a revised theoretical LIDT ratio of 1.2. Extreme scenario analysis demonstrates this ratio’s sensitivity to measurement errors: when assuming accurate LIDT_20%_ (5740 W/cm) but underestimated LIDT_40%_ (4783 W/cm), the model predicts LIDT_90%_=4237 W/cm. Conversely, with overestimated LIDT_20%_ (4924 W/cm) but accurate LIDT_40%_ (4103 W/cm), it predicts LIDT_90%_=3644 W/cm. The experimental value of LIDT_90%_ (3872 W/cm) falls precisely within this prediction band, conclusively attributing the original 17.8% LIDT_90%_ prediction error to measurement uncertainties in the LIDT.

## Conclusion

The laser damage mechanism of double-sided coated narrow-band filters based on RG-850 colored glass, subjected to CW laser irradiation at 1080 nm, is investigated in this study. The observed ablation perforation follows a three-stage process: gradual heat accumulation, nonlinear absorption intensification triggered by a critical temperature and ultimate vaporization ablation. This damage is primarily attributed to substrate absorption rather than coating degradation, as confirmed by instantaneous perforation morphology and the absence of progressive film damage. The multiple reflection model establishes the relationship between coating structure and substrate energy deposition, enabling a parameter inversion method that derives an initial equivalent average absorption coefficient of 0.4/cm from experimental LIDT ratios of two transmittance cases (20% vs. 40%), achieving LIDT predictions for arbitrary transmittance with a maximum deviation of 17.8%. Subsequent finite element analyses validate and refine this approach, yielding an optimized coefficient of 0.17/cm that reduces the LIDT prediction errors to 7.3%, with uncertainty analysis attributing the initial discrepancy to LIDT measurement uncertainties.

## Data Availability

The datasets used or analysed during the current study available from the corresponding author on reasonable request.
